# Sub-hypnotic dose of propofol as antiemetic prophylaxis attenuates intrathecal morphine-induced postoperative nausea and vomiting, and pruritus in parturient undergoing cesarean section — a randomized control trial

**DOI:** 10.1186/s12871-019-0847-y

**Published:** 2019-09-14

**Authors:** Sylvanus Kampo, Alfred Parker Afful, Shiraj Mohammed, Michael Ntim, Alexis D. B. Buunaaim, Thomas Winsum Anabah

**Affiliations:** 1 Department of Anesthesia and Intensive Care, School of Medicine and Health Science, University for Development Studies, Tamale, Ghana; 2 Department of Anesthesia, Tamale Teaching Hospital, Tamale, Ghana; 3 Department of Physiology, Dalian Medical University, Dalian, China; 4 Department of Surgery, School of Medicine and Health Science, University for Development Studies, Tamale, Ghana

**Keywords:** Antiemetic, Prophylaxis, Propofol, Metoclopramide, Postoperative nausea and vomiting, Pruritus, Parturient, Cesarean section

## Abstract

**Background:**

Postoperative Nausea and Vomiting (PONV) is a dreadful and uncomfortable experience that significantly detracts patients’ quality of life after surgery. This study aimed to examine the antiemetic effect of a single sub-hypnotic dose of propofol as prophylaxis for PONV.

**Method:**

In this prospective, double-blind, randomized control trial, 345 parturients presented for elective cesarean section at the Obstetric unit of Tamale Teaching Hospital were recruited. Each recruited parturient was randomly assigned to one of three groups; Propofol group (*n* = 115) represented those who received propofol 0.5 mg/kg, Metoclopramide group (*n* = 115) represented those who received metoclopramide 10 mg and, Control group (*n* = 115) represented those who received 0.9% saline. Spinal anesthesia with 0.5% hyperbaric bupivacaine 7.5–10 mg, and intrathecal morphine 0.2 mg was employed for the anesthesia.

**Results:**

The data indicate that 108 (93.9%) parturients from the control group, 10 (8.7%) from the propofol group and 8 (7.0%) from the metoclopramide group experienced some incidence of PONV. There was no significant difference in the incidence of PONV (nausea, vomiting, and none) between the propofol and the metoclopramide groups (*P* = 0.99; 0.31; and 0.35 respectively). Parturients who received antiemetic agents were 105 (97.2%), 1 (10.0%) and 3 (37.5%) from the control, propofol and metoclopramide groups respectively. The data indicated that 98 (85.2%) parturients from the control, 3 (2.6%) from propofol group, and 100 (87.0%) from the metoclopramide group experienced some levels of pruritus. There was a significant difference in the incidence of pruritus (mild, moderate, and no pruritus) between the metoclopramide and propofol groups (*P* <  0.01; *P* <  0.01; and *P* <  0.01 respectively).

**Conclusion:**

A sub-hypnotic dose of propofol is effective as metoclopramide in the prevention of PONV in parturient undergoing cesarean section under spinal anesthesia with intrathecal morphine. Sub-hypnotic dose of propofol significantly reduces the incidence of postoperative pruritus following intrathecal morphine use.

**Trial registration:**

Current control trial, registered at ISRCTN trial registry: ISRCTN15475205. Date registered: 03/04/2019. Retrospectively registered.

## Background

Cesarean section is among the most commonly performed surgeries in women, and it is associated with a more intense postoperative pain compared to the post-vaginal delivery pain [1]. Excellent postoperative analgesia is very crucial in providing maternal comfort, improving breastfeeding, improving mother-child bonding, early ambulation, early discharge, and enhancing patient satisfaction [2]. The use of spinal anesthesia for cesarean section provides an avenue for rendering better postoperative analgesia with neuraxial opioids [1]. Intrathecal morphine provides excellent postoperative analgesia. The current practice of using spinal anesthesia with morphine for parturient presenting for elective cesarean delivery in some hospitals has received mixed reactions from both parturient and staff. Although the addition of intrathecal morphine to bupivacaine offers excellent postoperative analgesia which covers for about 12 to 24 h, some fraction of parturients commonly experiences dose-dependent PONV and pruritus [3].

PONV is an unpleasant condition, often underestimated side effect of anesthesia and surgery [4]. Despite the increasing fear of pain after surgery, patients still consider PONV to be a significant concern or complication of anesthesia [5]. When questioned about issues of concern, 22% of 800 patients in a study gave PONV the highest level of concern compared with 34% for postoperative pain and 24% for waking up during surgery [6]. Gan et al. [7] reported that most patients associated value to the avoidance of PONV and were willing to pay between the US $56 and the US $100 for a completely effective antiemetic. Due to its medical, surgical, patient and anesthetic etiological factors, its incidence is estimated to be 40 to 60% of all surgical interventions and patient population of which, 0.18% is resistant to PONV [8]. The intense efforts accompanying PONV increases the risk of aspiration pneumonitis, wound dehiscence, bleeding, hypertension, and increased intracranial pressure [9]. It also leads to higher consumption of calories, requires additional postoperative monitoring, and delayed discharge leading to a higher cost of care [10]. Other morbidities synonymous with PONV also includes; dehydration, electrolyte disturbance, interference with nutrition and, more rarely, esophageal rupture [11].

Prophylaxis with antiemetic has been shown to reduce the incidence of PONV in surgical procedures by 15–30% (absolute risk reduction) [12]. Numerous antiemetic has been studied for the prevention of PONV with varying degrees of success [13]. The efficacy of metoclopramide as an antiemetic is undoubted. Propofol anesthesia is known to have a low emetic score, and its antiemetic properties have been investigated. While it was found to be effective by some studies, the contrary was reported in some other studies [8]. Series of clinical trials have also reported that, at a sub-hypnotic dose, propofol is equally effective in reducing the incidence of not only PONV but also pruritus following intrathecal morphine [14]. Although routine PONV prophylaxis seems appropriate, the choice of antiemetic agents is wide, whereas some are too expensive in our setting for regular use. This study, therefore, aimed to ascertain the antiemetic effect, as well as reducing pruritus by a sub-hypnotic dose of propofol and compare its effect with metoclopramide among parturients receiving neuraxial morphine for cesarean section.

## Methods

### Ethical statement

This double-blind, randomized control trial was carried out at the Tamale Teaching Hospital from April 2016 to May 2017. The ethical committee of the Tamale Teaching Hospital approved the study protocol (ID No: TTHERC21/04/16/08). The clinical trial registration number is ISRCTN15475205. The study protocol adhered to the CONSORT guidelines. Written informed consent was obtained from individual parturient after providing them with adequate explanations regarding the aims of the study.

### Subjects

This study recruited three hundred and sixty (360) parturients. The inclusion criteria were as follows: Parturients with gestational age ≥ 36 weeks who reported at the obstetric unit of the Tamale Teaching Hospital and were scheduled to undergo elective cesarean section under spinal anesthesia with intrathecal injection of morphine, age 20 to 40 years old, American Society of Anesthesiologists Physical Status (ASA-PS) score 1–2. The exclusion criteria were as follows: parturients who did not give consent, or have history of nausea and vomiting before pregnancy, relevant drug allergy, any co-morbidity, motion sickness, abdominal surgery or experienced intraoperative blood loss (EBL) ≥ 500 mL during surgery.

### Randomization

**Fig. 1:**
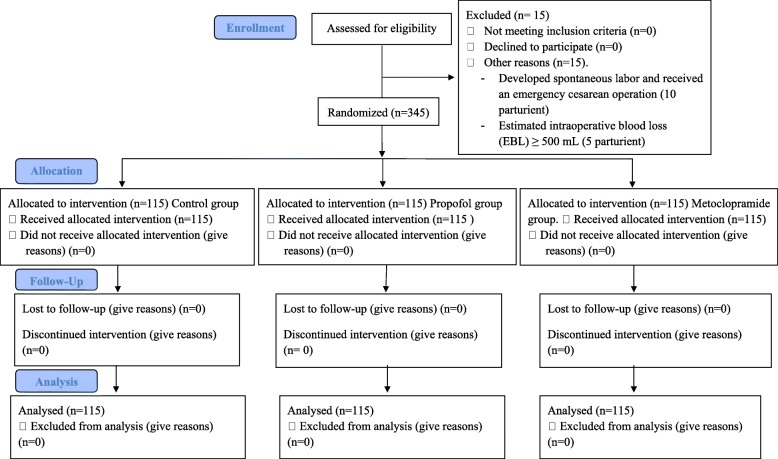
CONSORT recommended description for patient recruitment

Each recruited parturient was randomly assigned to one of three groups using a computer-generated random number table. The group allocation was concealed in a sealed opaque envelope which was opened just before the administration of the drugs 10–15 min before the end of surgery. Propofol group (*n* = 115) represented those who received intravenous propofol (0.5 mg/kg), Metoclopramide group (*n* = 115) represented those who received intravenous metoclopramide (10 mg) and, Control group (*n* = 115) represented those who received intravenous saline (0.9%) as negative control (Fig. 1).

### Drugs

The sources of drugs were as follows; 1% propofol (10LF2786, South Africa), metoclopramide (171A-080, Imres), 0.5% hyperbaric bupivacaine (F0223–1, AstraZeneca, UK), preservative-free 2% lidocaine (P7445, Layina pharmaceuticals PYT, LTD, India), morphine (P7445, Layina pharmaceuticals PYT, LTD, India), Suppository diclofenac (P7445, Layina pharmaceuticals PYT, LTD, India), Tramadol (P7445, Layina pharmaceuticals PYT, LTD, India).

### Anesthesia induction and drug application

All parturients were prospectively assessed and classified according to the America Society of Anesthesiologist (ASA) physical status classification. Basic intraoperative monitoring (ECG, SpO2_,_ Temperature, and non-invasive blood pressure) were applied, and the baseline vital signs checked and recorded. All recruited parturients had no history of nausea or vomiting 72 h before surgery. Before surgery, the individual parturient was advised not to eat any solid food for at least 6–8 h. An independent anesthesiologist, specialized in obstetric anesthesia was assigned to perform the spinal anesthesia and monitor the parturient till discharged from the hospital.

In the sitting position, the skin and interspinous ligaments were infiltrated with 2 ml of preservative-free 2% lidocaine using a 21G hypodermic needle. Lumbar puncture was then performed aseptically using a 26G pencil point spinal needle by the midline approach at the lumbar region (L2-L3 or L3-L4 interspace). Successful insertion of the spinal needle into the subarachnoid space was confirmed by the presence of the free flow of cerebrospinal fluid. The subarachnoid block was then established with 7.5–10 mg preservative-free hyperbaric bupivacaine, and 0.2 mg morphine. The individual parturient was then asked to return to the supine position with their head supported on a pillow and slightly tilted up to avoid any further spread of the spinal agent toward the head. A left lateral tilted for uterine displacement was employed to prevent aortocaval compression. The vital signs (pulse rate, blood pressure, oxygen saturation, and respiratory rate) of the individual parturient was monitored and recorded for every 5 min for the first 30 min and then for every 15 min. Ice cube was used to confirm adequate sensory block up to T_6_ level. Supplemental oxygen was given at 3 L/minutes through nasal prongs. Intraoperative hypotension was treated with 5–20 mg of intravenous ephedrine. Any estimated fluid deficit or blood lost was replaced accordingly. After the delivery of the baby, 5–10 unit of intravenous oxytocin was given to aid uterine contraction. An independent anesthesiologist who was blinded to drugs administration was asked to administer; saline (0.9%), metoclopramide 10 mg or propofol 0.5 mg/kg, 10–15 min before to the end of surgery.

### Measurements

The episodes of PONV were identified by direct scheduled assessments or by a spontaneous complaint by the patients after the surgery. The incidence of PONV was recorded hourly for the first 4 h and then 4 hourly for the next 24 h using a 3 point ordinal scale (0 = none, 1 = nausea, 2 = vomiting). The incidence of PONV was calculated and categorized as early (0–4 h) or delayed (5 – 24 h). Intravenous Kytril 1–2 mg (anti-emetic) was administered if nausea or vomiting ensues or on request. The proportion of parturient and the number of times they required rescue anti-emetic in each group were recorded.

Pain intensity was measured immediately after the surgery on a 100-mm VAS [15], 0 mm = no pain, and 100 mm = intolerable pain. If rescue analgesia was required, parturient received suppository diclofenac 100 mg or injection tramadol 100 mg or both. The incidence of pruritus was recorded every 4 h for 48 h after surgery on a four-point categorical scale as; 0 = no pruritus, 1 = mild, 2 = moderate, 3 = severe pruritus. Naloxone hydrochloride 2 μg/kg was injected to manage opioid depression, and Cetirizine 10 mg was administered if pruritus ensues or on request. Overall perioperative satisfaction was evaluated on the day of discharged during an interview as; 4 = excellent, 3 = good, 2 = satisfactory, 1 = poor.

### Statistical analysis

Due to the unknow of our population size, the sample size for this study was calculated using the equation;

$$ \mathrm{Necessary}\ \mathrm{Sample}\ \mathrm{Size}={\left(\mathrm{Z}\hbox{-} \mathrm{score}\right)}^2\ast \mathrm{StdDev}\ast \left(1\hbox{-} \mathrm{StdDev}\right)/{\left(\mathrm{margin}\ \mathrm{of}\ \mathrm{error}\right)}_2 $$

95% confidence interval (Z-score = 1.96), Standard Diviation (*StdDev = 0.5)* and margine of error = ± 5 or 6%. Therefore, our sample size adjustment was between 267 and 384 respondents.

All statistical analyses were carried out using the GraphPad Prism v 7.01 (GraphPad Software, La Jolla, CA, US). Statistical analysis was performed for age, weight, BMI, parity, gestational age, degree of hypotension, dose of ephedrine administered, exteriorization of uterus, duration of surgery, incidence and pattern of PONV, request for rescue antiemetic therapy, the incidence and pattern of pruritus, the use of rescue pain relief, and patient satisfaction of anesthesia service, using one-way ANOVA and multiple comparison by Tukey’s test. The student’s t-test was used for statistical comparisons between two groups. All values are depicted as mean and considered significant if *P* <  0.05.

## Results

Of the 360 parturients that were recruited for this study, data for 15 parturients were excluded from the analysis (10 parturients experienced spontaneous labor and received an emergency cesarean operation and 5 parturients estimated intraoperative blood loss (EBL) was greater than 500 mL). Therefore, data for 345 parturients comprising 115 each for control, propofol, and metoclopramide groups were included in the analysis (Fig. 1).

The data showed that there was no significant difference among parturients from the control group, propofol group, and the metoclopramide group regarding age, weight, BMI, primip parity, multiparity, grand multiparity and gestational age (*P* value = 0.73; *P* value = 0.92; *P* value = 0.78; *P* value = 0.91; *P* value = 0.49; *P* value = 0.91; and *P* value = 0.61 respectively) (Table 1).

**Table 1:** Demographic characteristics of respondents. Data were statistically significant at *P* <  0.05 compared with control

Measurements	Control (*n* = 115) Mean ± SD	Propofol (*n* = 115) Mean ± SD	Metoclopramide (*n* = 115) Mean ± SD	*P* Value
Age (years)	31.17 ± 5.12	33.33 ± 5.28	31.33 ± 5.35	0.73 (NS)
Weight (kg)	64.50 ± 7.23	64.83 ± 6.49	65.00 ± 7.10	0.92 (NS)
BMI	29.17 ± 3.49	30.50 ± 3.08	29.67 ± 3.27	0.78 (NS)
Primip parity	42.00 ± 0.58	42.00 ± 1.00	42.33 ± 1.53	0.91 (NS)
Multiparity	67.68 ± 0.58	67.00 ± 1.00	67.68 ± 0.58	0.49 (NS)
Grand Multiparity	5.67 ± 0.58	6.00 ± 0.00	6.00 ± 1.73	0.91 (NS)
Gestational age (weeks)	39.17 ± 1.72	38.83 ± 1.17	39.67 ± 1.37	0.61 (NS)

*BMI* Basal metabolic index, *n* Number of respondents included in the analysis, *SD* Standard deviation, *NS* No significant

The degree of hypotension experienced during the intraoperative period after the subarachnoid blockade indicated that 84 (73.0%) parturients from the control group, 76 (66.1%) from the propofol group, and 89 (77.4%) from the metoclopramide group experienced no degree of hypotension compared with baseline blood pressure. While 19 (16.5%) parturients from the control group, 27 (23.5%) from the propofol group, and 15 (13.0%) from the metoclopramide group experienced 10–20% decreased in blood pressure compared with the baseline blood pressure (Table 2). 8 (7.0%) parturients from the control group, 9 (7.8%) from the propofol group, and 10 (8.7%) from the metoclopramide group experienced 21–31% decreased in blood pressure compared with the baseline blood pressure. Also, 4 (3.5%) parturients from the control group, 3 (2.6%) from the propofol group, and 1(0.9%) from the metoclopramide group experienced 31–40% decreased in blood pressure when compared with the baseline blood pressure (Table 2). The results indicated that there was no significant difference among parturients from the propofol group, metoclopramide and the control group regarding 0%, 10–20%, and 31–40% decreased in blood pressure (*P* <  0.01; *P* <  0.01; *P* <  0.05 respectively). However, the 21–31% decreased in blood pressure showed no significant difference between the groups (Table 2). Hypotension caused by the subarachnoid block in individual parturient responded to ephedrine (5–20 mg) treatment. The doses of ephedrine administered indicated significant difference between the groups (*P* <  0.01; *P* <  0.01; *P* <  0.02) (Table 2). The duration of surgery ranged from 25 to 90 min and showed significant difference between the groups (*P* <  0.01; *P* <  0.01; *P* <  0.01) (Table 2). No episode of intraoperative emetic was noted for the individual groups.

**Table 2:** Intraoperative management. Paturient were monitored intraoperatively for degree of hypotension caused by the intrathecal injection of local anesthetic; the doses of ephedrine administered to manage the hypotension; exteriorization of uterus; and the duration of surgery. Data were statistically significant at *P* <  0.05 compared with control

Measurements	Control (*n* = 115)		Propofol (*n* = 115)		Metoclopramide (*n* = 115)		*P* Value
	Frequency	Percentage (%)	Frequency	Percentage (%)	Frequency	Percentage (%)	
Degree of hypotension (percentage decrease in blood pressure compared with the baseline blood pressure)							
0%	84	73.0	76	66.1	89	77.4	< 0.01
10–20%	19	16.5	27	23.5	15	13.0	< 0.01
21–30%	8	7.0	9	7.8	10	8.7	0.07 (NS)
31–40%	4	3.5	3	2.6	1	0.9	< 0.05
Dose of ephedrine (mg) administered							
0	84	73.0	76	66.1	89	77.4	< 0.01
5–10	27	23.5	36	31.3	25	21.7	< 0.01
11–20	4	3.5	3	2.6	1	0.9	< 0.02
Exteriorization of Uterus							
Yes	112	97.4	97	84.4	103	89.6	< 0.01
No	3	2.6	18	15.7	12	10.4	< 0.01
Duration of surgery (minutes)							
< 30	2	1.7	4	3.5	1	0.9	< 0.01
40–60	101	87.8	68	59.1	69	60.0	< 0.01
61–90	12	10.4	43	37.4	45	39.1	< 0.01

*NS* No significant, *n* Number of respondents included in the analysis

### Sub-hypnotic dose of propofol prevents morphine-induced PONV in cesarean section

**Fig. 2:**
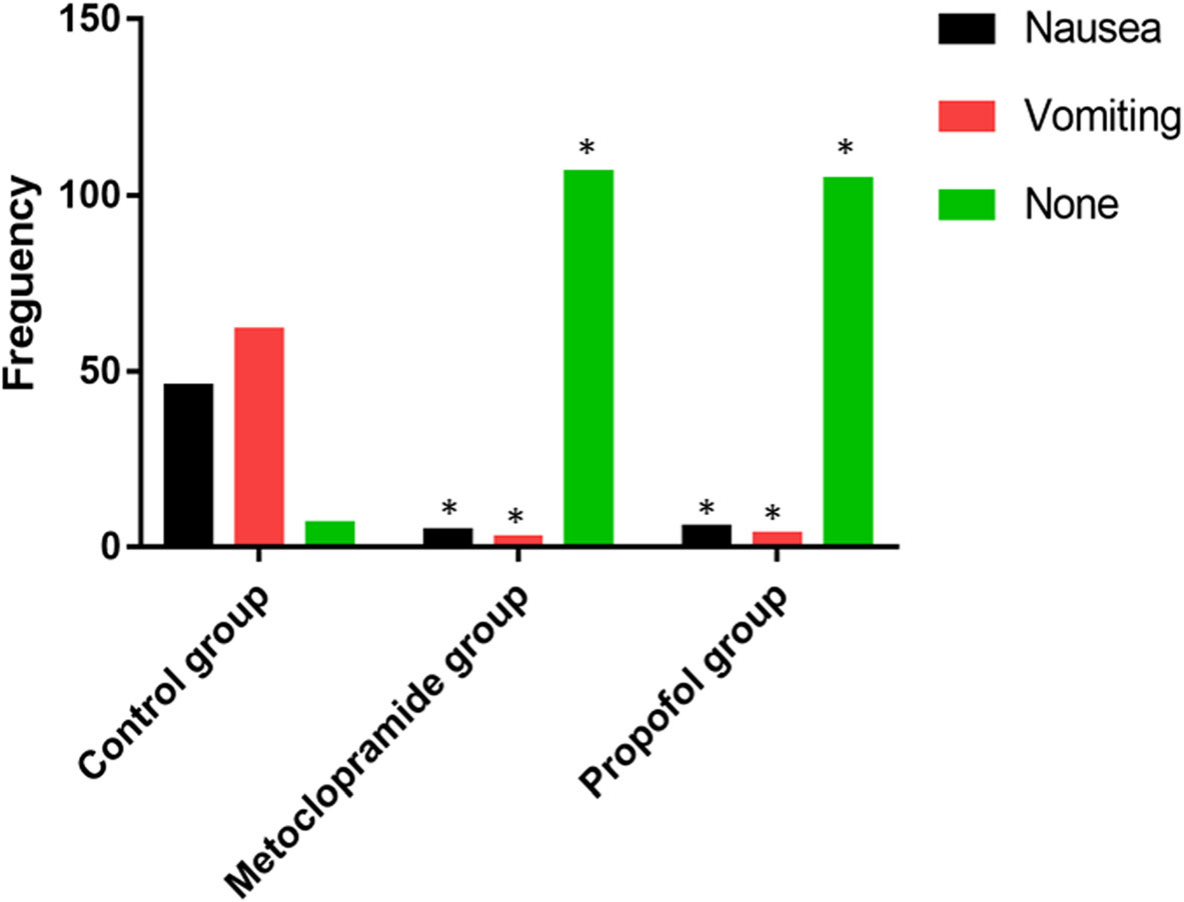
Incidence of PONV. The data correspond to the mean ± SD and was statistically significant at * indicates P <  0.05 compared with control

To investigate the antiemetic prophylaxis effect of propofol in cesarean section, we injected saline, propofol, or metoclopramide 10–15 min before the end of surgery. We then monitored parturients for any incidence of PONV for 24 h postoperatively. The data indicated that 108 (93.9%) from the control group, 10 (8.7%) from the propofol group, and 8 (6.9%) from the metoclopramide group experienced some levels of PONV (Fig. 2; Table 3). It was noted that the incidence of PONV significantly decreased in the propofol group compared with the control group (*P* <  0.01). Similarly, PONV significantly reduced in the metoclopramide group compared with the control group (*P* <  0.01) (Table 3). However, the data showed no significant difference in the incidences of PONV (nausea, vomiting and none) between the propofol and the metoclopramide groups (*P*-values = 0.99; *P*-values = 0.31; *P*-values = 0.35 respectively) (Fig. 2). It was also noted that 105 (97.2%) parturients from the control group, 1 (10.0%) from the propofol group, and 3 (37.5%) from the metoclopramide group received additional rescue antiemetic (Table 3). The data also indicated a significant difference between the groups for the rescue antiemetic therapy (*P* <  0.001; *P* <  0.01) (Table 3). This evidence, therefore, suggested that a low dose of propofol for antiemetic prophylaxis may equally be effective as metoclopramide in preventing intrathecal morphine-induced PONV in cesarean section.

**Table 3:** Pattern of PONV and request for rescue antiemetic therapy. The data were statistically significant at *P* < 0.05 compared with control

Measurements	Control (*n* = 115)		Propofol (*n* = 115)		Metoclopramide (*n* = 115)		*P* Value
	Frequency	Percentage (%)	Frequency	Percentage (%)	Frequency	Percentage (%)	
Pattern of PONV							
Early (0–4 h)	95	88.0	2	20.0	2	25.0	< 0.01
Late (4–24 h)	13	12.0	8	80.0	6	75.0	< 0.01
Request for rescue antiemetic therapy							
Yes	105	97.2	1	10.0	3	37.5	< 0.01
No	3	2.8	9	90.0	5	62.5	< 0.01

*PONV* Postoperative nausea and vomiting, *n* Number of respondents included in the analysis

### Sub-hypnotic dose of propofol prevents intrathecal morphine-induced postoperative pruritus

**Fig. 3:**
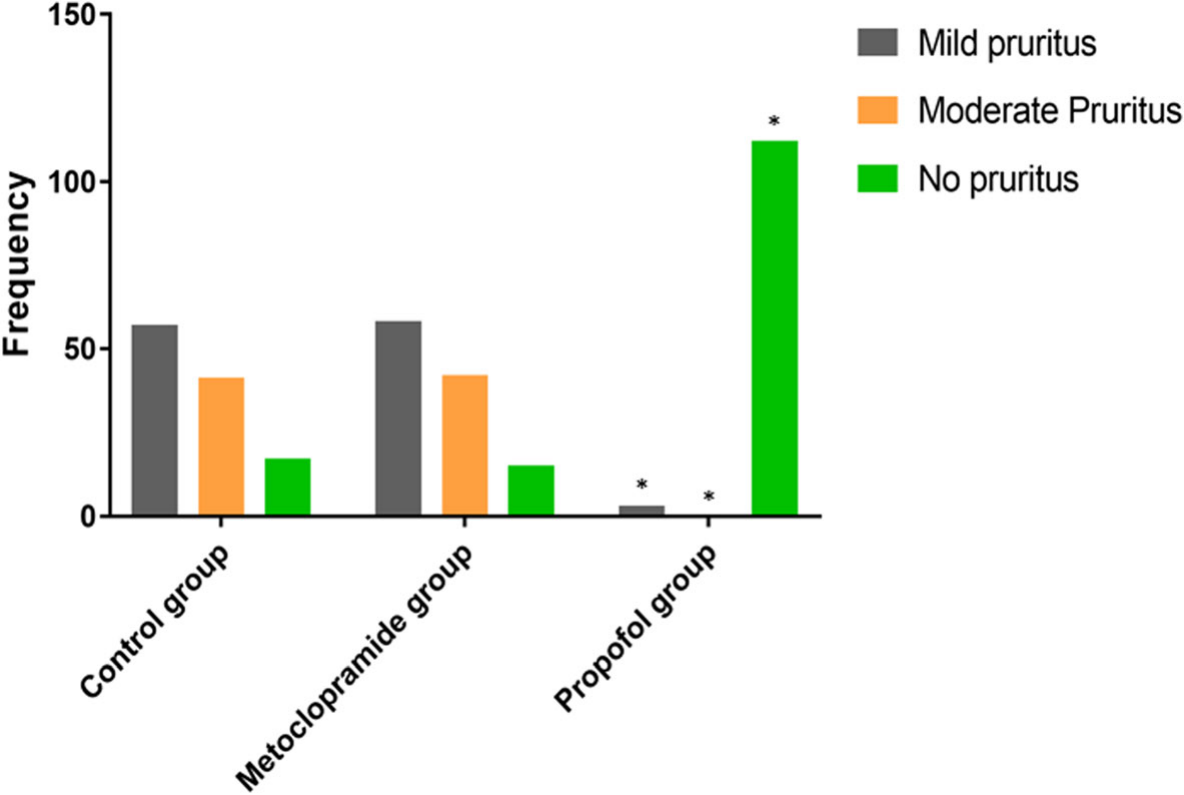
Propofol prevents morphine-induced pruritus. The data correspond to the mean ± SD and was statistically significant at * indicates P <  0.05 compared with control

In this study, we also determined the incidence of postoperative pruritus caused by the intrathecal injection of morphine. The results showed that 98 (85.2%) from the control, 3 (2.6%) from the propofol group, and 100 (87.0%) from the metoclopramide group experienced some levels of postoperative pruritus (Fig. 3). We observed that the sub-hypnotic dose of propofol significantly decreased the incidence of postoperative pruritus compared with metoclopramide (*P* <  0.01). We also observed that there was no significant difference in the incidence of pruritus between the metoclopramide and the control groups (*P* = 0.99). However, there was a significant difference in the incidence of pruritus between the propofol and the control groups (*P* <  0.01) (Fig. 3). The data also indicated that there were significant differences in the incidence of pruritus (mild, moderate, and no pruritus) between the metoclopramide and propofol groups (*P* <  0.01; *P* <  0.01; and *P* <  0.01 respectively) (Fig. 3). This evidence suggested that sub-hypnotic dose of propofol for antiemetic prophylaxis also exhibits a therapeutic effect against postoperative pruritus.

The data also showed that 114 (99.1%) parturients from the control group, 115 (100.0%) from the propofol group, and 114 (99.1%) from the metoclopramide group received no supplementary postoperative analgesia, whereas, 1 (0.9%) from the control group, 0 (0.0%) from the propofol group, and 0 (0.0%) from the metoclopramide group received suppository diclofenac (100 mg) as supplementary analgesia at the postoperative period (Table 4). There was no significant difference in the request for postoperative rescue analgesic (Supp. diclofenac, I.V. Tramadol, and None) between the individual groups (*P*-value = 0.13 for the control group, *P*-value = 0.22 for the propofol group and *P*-value = 0.73 for the metoclopramide group respectively) (Table 4). These findings, therefore, suggested that intrathecal injection of morphine in parturients undergoing cesarean section may provide adequate postoperative analgesia.

**Table 4:** Postoperative request for supplementary analgesic for pain and patient satisfaction of the anesthesia service. Data were statistically significant at *P* < 0.05 compared with control

Measurements	Control (*n* = 115)		Propofol (*n* = 115)		Metoclopramide (*n* = 115)		*P* Value
	Frequency	Percentage (%)	Frequency	Percentage (%)	Frequency	Percentage (%)	
Rescue pain relief administered							
Supp. diclofenac (100 mg)	1	0.9	0	0.0	0	0.0	0.13 (NS)
I.V. Tramadol (50 mg)	0	0.0	0	0.0	1	0.9	0.22 (NS)
None	114	99.1	115	100.0	114	99.1	0.73 (NS)
Patient satisfaction							
Excellent	9	7.8	93	80.9	89	77.4	< 0.01
Good	15	13.0	20	17.4	23	20.0	< 0.01
Satisfactory	45	39.1	2	1.7	3	2.6	< 0.01
Poor	46	40.0	0	0.0	0	0.0	< 0.01

*Supp.* Suppository, *I.V.* Intravenous, *n* Number of respondents included in the analysis, *NS* No significant

We next assessed the parturients satisfaction level of the anesthesia service. The data indicated that 9 (7.8%) parturients from the control group, 93 (80.9%) from the propofol group and 89 (77.4%) from the metoclopramide group scored excellent for anesthesia service. 15 (13.0%) parturients from the control group, 20 (17.4%) from the propofol group, and 23 (20.00%) from the metoclopramide group scored good for the anesthesia service. 45 (39.13%) respondents from the control group, 2 (1.7%) from the propofol group and 3 (2.6%) from the metoclopramide group scored satisfactory for the anesthesia, while 46 (40.0%) from the control group and none from propofol or metoclopramide groups scored poor for anesthesia service (Table 4). The data showed significant difference among parturient from the propofol or metoclopramide group regarding those who scored excellent, good, Satisfactory or poor for the anesthesia service compared with the control group (*P* <  0.01; *P* <  0.01; *P* <  0.01; *P* <  0.01 respectively) (Table 4). This emerging evidence, therefore, suggested that intrathecal injection of morphine with a sub-hypnotic dose of propofol for parturients undergoing cesarean section may improve postoperative analgesia and prevent intrathecal morphine-induced PONV and pruritus without compromising anesthetic reliability.

## Discussion

Obstetrics and gynecological surgeries, including cesarean section, are associated with the incidence of PONV as high as 60–83% most especially when no prophylactic antiemetic is provided [16]. This may be due to multiple complex factors such as; stimulation of uterus, broad ligament, vagina, and cervix, which may induce vomiting through afferents signals to the spinal cord along hypogastric and pelvic plexus. Surgical pain increases the circulating catecholamines, which cause PONV by stimulating area poster. Other nonanesthetic causes include surgical bleeding, medications, such as antibiotics and early motion at the end of surgery or history of motion sickness. Few anesthetic causes of PONV include hypotension, increased vagal activity, administration of neuraxial or parenteral opioids, and the addition of phenylephrine or epinephrine to local anesthetics. Also, peak block height ≥ T_5_, use of procaine, baseline heart rate ≥ 60 beats/min.

This study was designed to test the hypothesis that propofol use as antiemetic prophylaxis prevents intrathecal morphine-induced postoperative nausea and vomiting, as well as pruritus in parturients undergoing a cesarean section. The following principal observation emerged: First, the data indicated that sub-hypnotic dose of propofol was equally effective as metoclopramide in the prevention of PONV in parturients undergoing cesarean section under spinal anesthesia with intrathecal morphine. Second, the data showed that sub-hypnotic dose of propofol significantly reduced the incidence of postoperative pruritus following intrathecal morphine used. Some variables in this current study were kept constant for all study groups; the type of surgery, anesthesia technique, anesthetic drugs, and the level of the spinal block was all standardized, including postoperative analgesics. Duration of anesthesia and surgery were comparably the same, and there was no significant difference between age, weight, and BMI of patients from individual study groups. These, therefore, suggested that the significant difference in incidence and severity of PONV between study groups were solely attributed to the drugs tested.

Factors noted to induce emesis during cesarean delivery under spinal anesthesia includes; peritoneal traction, exteriorization of the uterus, fundal pressure during delivery of the baby and hypoxia associated with hypotension following spinal anesthesia. Pusch et al. [17] noted that emetic symptoms were reduced in patients who developed post-spinal hypotension after being given 100% oxygen, thus, implicating hypoxemia at the emetic center as a probable causative factor. This study recorded no significant changes between the groups concerning maternal blood pressure, pulse rate, respiratory rate, and oxygen saturation. The amount of ephedrine used for the treatment of hypotension was also similar between the groups. No intraoperative emesis was recorded from the study groups.

Metoclopramide is an inexpensive generic drug. As a benzamide, it acts on Dopamine 2 receptor to prevent the stimulation of the vomiting center. Its effectiveness as prophylaxis has also been confirmed [18]. Propofol is well known for its role in decreasing the incidence of PONV when used at a sub-hypnotic dose. However, the exact mechanism by which propofol prevents emesis is unknown. It has been postulated to be an antagonist at the 5HT3 receptor. Other reports suggest that the antiemetic effect of propofol is due to modulation of the subcortical pathways [19]. Patients who received propofol experienced a significant reduction in nausea and vomiting compared with patients treated with placebo. A survey demonstrated that 86% of patients who received a sub-hypnotic dose of propofol recorded no symptoms of emetic after surgery [20]. Emerging evidence also indicates that propofol, given at sub-hypnotic dose significantly decreases the incidence of emetic episodes in patients undergoing cesarean delivery with spinal anesthesia. In a study by Song et al. [16] it was demonstrated that propofol given after sevoflurane and desflurane anesthesia for outpatient laparoscopic cholecystectomy significantly decreased the incidence of PONV compared with control. Similarly, reports suggest that low-dose of propofol (0.5 or 1 mg/kg) administration at the end of surgery effectively reduce the incidence of PONV within 2 h postoperatively in highly susceptible women undergoing a laparoscopy-assisted vaginal hysterectomy and receiving opioid-based PCA [21]. In this present study, it was realized that 105 (91.30%) from the propofol group experienced no incidence of PONV compared with 7 (6.09%) parturient from the control group, a similar observation Chatterjee et al. [22], Apfel et al. [23] and Warltier et al. [24] have also earlier submitted. Comparing the episodes and severity of PONV, the data from this study suggested that parturient who received metoclopramide (10 mg) experienced less incidence of PONV than those who received sub-hypnotic dose of propofol (0.5 mg/kg). However, the used of rescue antiemetic was higher in the metoclopramide group compared with the propofol group.

Pruritus is one of the most common adverse effects of intrathecal morphine. It is most challenging to treat and respond poorly to conventional antihistamine treatment [25]. Therefore, it remains a significant challenge for the anesthesiologist. Existing reports indicate that a low dose of propofol could alleviate morphine-induced pruritus without disrupting intrathecal morphine analgesia [26–28]. In this study, it was noted that sub-hypnotic dose of propofol decreased the incidence of pruritus compared with metoclopramide, an observation Liu et al. [29] has earlier reported. This emerging evidence, therefore, suggested that a low dose of propofol as antiemetic prophylaxis attenuate not only PONV but also, morphine-induced pruritus.

## Conclusion

In conclusion, this study findings suggested that a sub-hypnotic dose of propofol could be as effective as metoclopramide in the prevention of PONV in parturient undergoing cesarean section under spinal anesthesia with intrathecal morphine. Also, a single sub-hypnotic dose of propofol may result in a low incident of opioid-induced pruritus. Therefore, propofol may be a better choice of antiemetic prophylaxis for opioid-induced PONV and pruritus in cesarean section.

## Data Availability

The datasets generated and/or analyzed during the current study are not publicly available due to patient confidentiality but are available from the corresponding author on reasonable request.

## Abbreviations

ASA: Americ Society of Anesthesiology
EBL: Estimated Blood Loss
ECG: Electrocardiogram
PONV: Postoperative nausea and vomiting
SpO2: Pulse Oximetry
VAS: Visual Analogue Scale
